# Качество жизни больных первичным гиперпаратиреозом до и в разные сроки после хирургического лечения и факторы, определяющие его улучшение

**DOI:** 10.14341/probl13386

**Published:** 2023-10-23

**Authors:** Т. И. Ионова, Р. А. Черников, И. В. Слепцов, Д. М. Бузанаков, С. М. Ефремов, Т. П. Никитина, И. С. Тюлюбаев, А. В. Золотухо, К. А. Бубнов, А. А. Виноградова, В. В. Скворцов, В. Ф. Русаков

**Affiliations:** Санкт-Петербургский государственный университет, Клиника высоких медицинских технологий им. Н. И. Пирогова; Санкт-Петербургский государственный университет, Клиника высоких медицинских технологий им. Н. И. Пирогова; Санкт-Петербургский государственный университет, Клиника высоких медицинских технологий им. Н. И. Пирогова; Санкт-Петербургский государственный университет, Клиника высоких медицинских технологий им. Н. И. Пирогова; Санкт-Петербургский государственный университет, Клиника высоких медицинских технологий им. Н. И. Пирогова; Санкт-Петербургский государственный университет, Клиника высоких медицинских технологий им. Н. И. Пирогова; Санкт-Петербургский государственный университет, Клиника высоких медицинских технологий им. Н. И. Пирогова; Санкт-Петербургский государственный университет, Клиника высоких медицинских технологий им. Н. И. Пирогова; Санкт-Петербургский государственный университет; Санкт-Петербургский государственный университет; Санкт-Петербургский государственный университет; Кафедра терапии (усовершенствования врачей), ФГБВОУ ВО «Военно-медицинская академия им. С.М. Кирова»; Санкт-Петербургский государственный университет, Клиника высоких медицинских технологий им. Н.И. Пирогова

**Keywords:** первичный гиперпаратиреоз, паратиреоидэктомия, качество жизни, симптомы

## Abstract

ОБОСНОВАНИЕ. Для комплексной оценки эффекта хирургического лечения у больных первичным гиперпаратиреозом (ПГПТ) актуальным является анализ закономерностей изменений качества жизни и симптомов при длительном мониторинге после паратиреоидэктомии (ПТЭ). ЦЕЛЬ. Цель данного исследования заключалась в изучении качества жизни больных ПГПТ до и в разные сроки после ПТЭ и определении факторов, связанных с его улучшением после операции.МАТЕРИАЛЫ И МЕТОДЫ. В рамках проспективного наблюдательного исследования все пациенты заполняли опросники до, через 3, 12, 24 мес и более после операции. Статистический анализ проводили с помощью t-критерия Стьюдента, U-теста Манна-Уитни в соответствии с характеристиками данных, критерия χ2 , метода обобщенных оценочных уравнений (generalized estimating equations, GEE) и бинарной логистической регрессии. Различия рассматривали значимыми при уровне p<0,05.РЕЗУЛЬТАТЫ. В исследование включены 82 пациента (средний возраст 53,7 года, 95% — женщины) с манифестной (73%) и бессимптомной (27%) формами заболевания. Медиана длительности наблюдения — 20 (3–31) мес. Через 3 мес после ПТЭ имело место значимое увеличение показателей качества жизни по всем шкалам общего опросника SF-36, кроме шкалы боли, по сравнению с их предоперационными значениями с последующим сохранением положительных изменений в отдаленные сроки после операции (GEE, p<0,001). Установлено существенное увеличение суммарного показателя, физического и психического компонентов по специальному опроснику PHPQoL после ПТЭ (GEE, p<0,05). В отдаленные сроки после операции показатели по всем шкалам SF-36, кроме ролевого физического функционирования (p=0,011), стали сопоставимы с группой сравнения, соответствующей группе пациентов по полу и возрасту (n=60, 52,5±9,2 года, 95% — женщины). Независимым предиктором значимого улучшения качества жизни после ПТЭ является предоперационный уровень психического компонента по опроснику PHPQoL (p=0,001) — чем ниже его уровень, тем больше вероятность значимого улучшения качества жизни по суммарному показателю PHPQoL после операции (ОШ=0,924, р=0,004).ЗАКЛЮЧЕНИЕ. ПТЭ сопровождается существенным улучшением качества жизни и регрессией симптомов у больных ПГПТ в течение длительного периода после операции. Независимым предиктором значимого улучшения качества жизни после ПТЭ является предоперационный уровень психологической составляющей качества жизни.

## Abstract (en)

BACKGROUND: For a comprehensive assessment of the effect of surgery in patients with primary hyperparathyroidism (PHPT) it sounds reasonable to evaluate quality of life (QoL) and symptoms in PHPT patients at long-term after parathyroidectomy (PTE). The purpose of this study was to study the quality of life of patients with PHPT before and at different times after PTE and to determine the factors associated with its improvement after surgery.MATERIALS AND METHODS: During prospective observational study, patients filled out QoL questionnaires before, 3, 12, 24 months or more after surgery. Statistical analysis was performed using the Student’s t-test, the Mann-Whitney U-test, χ2 criterion, the method of generalized estimating equations (GEE) and binary logistic regression. The differences were considered significant at the level of p<0.05.RESULTS: The study included 82 patients (mean age 53,7 years, 95% female) with symptomatic (73%) and asymptomatic (27%) PHPT. Median follow–up duration was 20 (3–31) months. At 3 months after PTE, there was a significant increase in QoL for all scales of the generic SF-36 questionnaire, except for the pain scale, compared with their preoperative values, followed by the preservation of positive changes at long term after surgery (GEE, p<0.05). At the long term after surgery, scores for all SF-36 scales, except for role physical functioning (p=0.011), became similar with the ones in comparison group adjusted to patients by gender and age (n=60, 52.5±9.2 years, 95% were women). An independent predictor of significant improvement in QoL after PTE was the preoperative level of the mental component according to the PHPQoL questionnaire (p=0.001) — the lower its level, the greater the probability of significant improvement in QoL according to the total PHPQoL index after surgery (OR=0.924, p=0.004).CONCLUSION. PTE is accompanied by significant improvement in QoL and regression of symptoms in patients with PHPT at long term follow-up after surgery. An independent predictor of significant QoL improvement after PTE is the preoperative level of the psychological component of QoL.

## ОБОСНОВАНИЕ

Первичный гиперпаратиреоз (ПГПТ) — распространенное эндокринное заболевание, характеризующееся избыточной автономной секрецией паратиреоидного гормона (паратгормона, ПТГ) при верхне-нормальном или повышенном уровне кальция крови одной или несколькими околощитовидными железами [1][2]. ПГПТ проявляется полиорганными нарушениями различной степени выраженности и может сопровождаться разнообразными симптомами, связанными с висцеральными и костными проявлениями, а также с нейро-когнитивными изменениями [3][4]. ПГПТ может протекать без диагностируемых традиционными методами висцеральных и костных изменений («бессимптомной») и с поражением костей и внутренних органов манифестной («симптомной») формах. Заболевание может приводить к существенному снижению качества жизни, инвалидизации пациентов, повышенному риску преждевременной смерти прежде всего от сердечно-сосудистых причин

В настоящее время паратиреоидэктомия (ПТЭ) является основным и самым эффективным методом лечения ПГПТ и показана всем пациентам с манифестной формой заболевания, пациентам моложе 50 лет при бессимптомной форме, при суточной экскреция кальция выше 10 ммоль или повышении уровня кальция в сыворотке крови на 0,25 ммоль/л. Хирургическое лечение может быть проведено также при бессимптомной форме ПГПТ и отсутствии явных общепринятых показаний к ПТЭ по требованию самого пациента, при этом необходима оценка соотношения риска/пользы от операции [6]. Кроме того, высокий уровне кальция крови может привести к развитию гиперкальциемического криза угрожающего жизни пациента что является абсолютным показанием к проведению ПТЭ при тяжелой гиперкальциемии [2][5].

Преимущества оперативного лечения заключаются в нормализации фосфорно-кальциевого обмена и устранении ассоциированных с гиперкальциемией симптомов, значимом улучшении состояния костной ткани и почек, сердечно-сосудистой системы и положительных изменениях когнитивных функций у абсолютного большинства прооперированных пациентов [5]. Как результат, ПТЭ приводит к улучшению качества жизни пациентов [7][8].

Качество жизни больного ПГПТ после ПТЭ характеризует эффект операции с точки зрения пациента и является одним из критериев эффективности оперативного лечения. В настоящее время имеются зарубежные данные, свидетельствующие о положительных изменениях разных аспектов качества жизни у больных ПГПТ после операции [7][9–12]. В этих работах проведен анализ степени восстановления разных аспектов функционирования пациентов после операции, изучены изменения качества жизни в разные сроки после хирургического лечения, в том числе, у пациентов с бессимптомным течением болезни и с манифестной формой ПГПТ, а также с разной степенью гиперкальциемии, определены предикторы улучшения качества жизни у больных после операции. Отечественных данных по динамике качества жизни у больных ПГПТ после ПТЭ крайне мало [13][14].

Ранее нами изучено качество жизни больных ПГПТ до оперативного вмешательства и проведен предварительный анализ изменений качества жизни пациентов после ПТЭ [15]. Также нами продемонстрирована целесообразность и информативность комплексной оценки качества жизни с применением комбинации общего и специальных опросников [16]. Рекомендуемыми специальными инструментами при ПГПТ являются опросник для оценки качества жизни больных ПГПТ — Primary Hyperparathyroidism Quality of Life (PHPQoL) и опросник оценки симптомов после паратиреоидэктомии — Parathyroidectomy Assessment of Symptoms (PAS) [16][17]. Общим опросником, который является информативным для оценки качества жизни при ПГПТ, является опросник SF-36. Комбинированное использование этих опросников может позволить выявить особенности изменения качества жизни у пациентов с ПГПТ до и после операции.

Остается актуальным изучение закономерностей изменений качества жизни и симптомов у больных ПГПТ при длительном мониторинге после ПТЭ и анализ факторов, которые оказывают влияние на положительные изменения качества жизни после операции, в том числе, изучение влияния исходного качество жизни до ПТЭ на его изменение в послеоперационный период. Данная информация может быть полезной для обоснования целесообразности оценки качества жизни и симптомов у пациентов с ПГПТ до операции и при последующем наблюдении после ПТЭ для мониторинга состояния пациентов в рутинной клинической практике, а также в выборе лечебной тактики у них.

## ЦЕЛЬ ИССЛЕДОВАНИЯ

Целью исследования являлось изучение качества жизни больных ПГПТ до и в разные сроки после ПТЭ и определение факторов, которые связаны с его улучшением после операции.

## МАТЕРИАЛЫ И МЕТОДЫ

## Место и время проведения исследования

Одноцентровое наблюдательное проспективное исследование проводили с сентября 2019-го по сентябрь 2023 гг. на базе отделения эндокринной хирургии Клиники высоких медицинских технологий им. Н.И. Пирогова СПбГУ, Санкт-Петербург.

## Методы

Критерии включения пациентов в исследование были следующими: 1) подтвержденный диагноз ПГПТ; 2) возраст ≥18 лет; 3) наличие показаний к хирургическому лечению в соответствии с современными клиническими рекомендациями [1]; 4) письменное информированное согласие пациента; 5) способность пациента заполнять опросники. Диагноз ПГПТ был подтвержден на основании результатов клинического, инструментального биохимического исследований. Все иные состояния, которые могли бы имитировать течение ПГПТ, были исключены при предоперационном обследовании. В исследование включали пациентов как с манифестной, так и бессимптомной формами ПГПТ. Манифестная форма характеризуется типичными клиническими проявлениями: специфическим для ПГПТ поражением почек (нефролитиаз, нефрокальциноз), скелета (остеопороз, фиброцистический остеит), верхних отделов желудочно-кишечного тракта (рецидивирующие язвы, панкреатит). Бессимптомная форма характеризуется подтвержденным прежде всего биохимически ПГПТ без скелетных и висцеральных проявлений с неспецифическими жалобами или без них, такими как усталость, бессонница, раздражение и т.д. Не включали в исследование пациентов с неспорадическим ПГПТ. Также критериями исключения являлись наличие выраженных проявлений сопутствующих заболеваний и психических расстройств. Для оценки уровня коморбидности использовали индекс коморбидности Чарлсон [18].

Всем пациентам была выполнена ПТЭ по стандартной методике с двусторонней ревизией шеи. Перед операцией проводили следующие исследования: определяли уровень ионизированного кальция (Ca2+), фосфора, ПТГ сыворотки крови, скорость клубочковой фильтрации, уровень витамина D, клиренс креатинина, суточную экскрецию кальция и фосфора с мочой, ЭКГ, а также выполняли клинический анализ крови и общий анализ мочи, остеоденситометрию. Выполнялись также общепринятые обязательные предоперационные исследования.

При выписке после ПТЭ всем пациентам были даны рекомендации по приему препаратов кальция, альфакальцидола или колекальциферола по показаниям под контролем показателей фосфорно-кальциевого обмена. В послеоперационный период уровни Ca2 +, ПТГ, 25OHD и другие биохимические параметры контролировались амбулаторно по месту жительства через 3 недели, 3 и 6 месяцев после операции. Пациентам были также даны рекомендации обращаться к специалистам клиники при возникновении каких-либо жалоб. Не было зарегистрировано ни одного случая обращения пациентов по поводу персистирующей/рецидивирующей гиперкальциемии, а также ни одного случая постоянной послеоперационной гипокальциемии.

Для оценки качества жизни и симптомов до операции все пациенты заполняли опросники на бумажных носителях, после операции — дистанционно, с помощью электронных форм через 3 месяца, 12 месяцев, 24 месяца и более после операции.

Для оценки качества жизни использовали общий опросник качества жизни RAND SF-36 и специальный опросник PHPQoL. Наличие и интенсивность симптомов анализировали на основании специального опросника оценки симптомов PAS.

RAND SF-36 — общий опросник, который может использоваться для оценки качества жизни здоровых людей и пациентов с хроническими заболеваниями [19]. Опросник состоит из 36 вопросов, которые формируют 8 шкал: физическое функционирование (ФФ), ролевое физическое функционирование (РФФ), боль (Б), общее здоровье (ОЗ), жизнеспособность (Ж), социальное функционирование (СФ), ролевое эмоциональное функционирование (РЭФ), психическое здоровье (ПЗ). Чем выше показатели по SF-36, тем лучше качество жизни. Для оценки изменения показателей качества жизни у пациентов с ПГПТ после операции проводили сравнение средних показателей по опроснику SF-36 у пациентов через 12 месяцев и более после операции со средними значениями показателей условно-здоровых респондентов того же пола и возраста. Для анализа использовали полученные ранее данные нормативных показателей качества жизни [20].

PHPQoL — специальный опросник качества жизни для пациентов с ПГПТ [16]. Он включает 16 вопросов, каждый из которых оценивается по шкале Ликерта в диапазоне от 0 до 4 (всегда, очень часто, время от времени, очень редко и никогда). Сумму баллов по шкалам Ликерта для 16 вопросов преобразуют с помощью процедуры стандартизации в суммарный показатель качества жизни, значения которого могут варьировать от 0 до 100 — чем выше суммарный показатель, тем лучше качество жизни. Формула для расчета суммарного показателя по опроснику PHPQoL представлена ниже.

Суммарный показатель= номинальное количество баллов по 16 вопросам/64 * 100.

Таким же способом рассчитывается физический компонент (ФК) качества жизни (стандартизированная сумма баллов по 9 вопросам опросника) и психический компонент (ПК) качества жизни (стандартизированная сумма баллов по 7 вопросам опросника).

ФК = номинальное количество баллов по 9 вопросам/36 * 100

ПК=номинальное количество баллов по 7 вопросам/28 * 100.

На основании суммарного показателя по опроснику PHPQoL определяли клинически значимое улучшение качества жизни больных в процессе лечения, которое соответствует увеличению суммарного показателя на 9 и более баллов по сравнению с его исходным значением [16]. Значимое улучшение качества жизни устанавливали в том случае, если хотя бы на одном сроке наблюдения регистрировали увеличение суммарного показателя PHPQoL на 9 и более пунктов по сравнению с показателем до операции.

Опросник PAS позволяет оценить 13 симптомов, распространенных при ПГПТ: усталость, жажда, перемены в настроении, боль в суставах, постоянная раздражительность, плохое настроение/депрессия, слабость, кожный зуд, забывчивость, головная боль, боль в животе, боль в костях, проблемы при вставании из положения сидя [17]. Симптомы количественно оценивали по шкале от 0 до 100, «0» обозначает полное отсутствие симптома, «100» — максимальную выраженность симптома, которую можно себе представить. Значение в диапазоне от 40 до 100 баллов рассматривали как значительно выраженный симптом. В исследовании оценивали все 13 симптомов опросника PAS согласно их выраженности от 0 до 100 баллов. Расчет единого показателя выраженности симптомов, предусмотренный в опроснике PAS, не проводили. Анализ динамики симптомов после операции проводили для тех симптомов, которые до операции встречались у более половины пациентов и имели выраженную интенсивность (40–100 баллов).

## Статистический анализ

Описательная статистика для непрерывных данных была представлена в виде количества наблюдений, средних арифметических значений и стандартных отклонений, медиан, диапазонов, межквартильных диапазонов и 95% доверительных интервалов (ДИ); для категориальных переменных данные были представлены в виде частот и долей. Характер распределения количественных переменных определяли с помощью критерия Шапиро-Уилка. Для статистических сравнений использовали t-тест Стьюдента, U-тест Манна-Уитни в соответствии с характеристиками данных и критерий χ².

Для оценки изменений качества жизни во времени после операции были применены обобщенные оценочные уравнения (GEE). Для изучения связи между параметрами до операции и того, испытал ли пациент значимое улучшение качества жизни после операции, применяли бинарную логистическую регрессию. Для проверки мультиколлинеарности между независимыми переменными использовали корреляции Спирмена. Все переменные, которые не имели сильной корреляции друг с другом (Spearman r<0,8), при уровне статистической значимости p<0,05 на этапе однофакторного анализа были включены в многомерный регрессионный анализ методом единовременного ввода. Результаты бинарной логистической регрессии представлены в виде отношения шансов (OШ) с 95% ДИ для каждого предиктора.

Все тесты были двусторонними с уровнем статистической значимости p<0,05. Статистический анализ проводился с использованием пакета прикладного программного обеспечения SPSS 23.0.

## Этическая экспертиза

Исследование одобрено Комитетом по биомедицинской этике Клиники высоких медицинских технологий им. Н.И. Пирогова СПбГУ (выписка из протокола №08/19 от 15.08.2019).

## РЕЗУЛЬТАТЫ

В анализ включено 82 больных ПГПТ. Средний возраст пациентов составил 53,7±10,2 года, 95% — женщины. Основные характеристики выборки представлены в таблице 1.

**Table:** Таблица 1. Характеристика выборки пациентов с ПГПТ ИМТ — индекс массы тела; Ca²⁺ — ионизированный кальций сыворотки; ПТГ — дооперационный уровень паратгормона плазмы крови; ПГПТ — первичный гиперпаратиреоз.

Характеристики	Значения
Общее количество, n (%)	82 (100)
Медиана возраста (диапазон), лет	55,5 (21–85)
Соотношение мужчины/женщины, n (%)	4 (4,9)/78 (95,1)
Образование, n (%)	
Среднее/среднее специальное	20 (24,4)
Высшее	62 (74,4)
Медиана ИМТ (диапазон), кг/м²	26,5 (19,3–45,4)
Медиана предоперационного уровня Ca²⁺ (диапазон), ммоль/л (нормальные значения 1,1–1,3)	1,45 (1,2–2)
Медиана предоперационного уровня ПТГ (диапазон), пмоль/л (нормальные значения 1,6–6,9)	14,9 (7,2–84,5)
Форма ПГПТ, n (%)	
Бессимптомная	22 (26,8)
Манифестная	60 (73,2)
Уровень гиперкальциемии, n (%)	
Незначительная (ионизированный кальций сыворотки ≤1,49 ммоль/л)	55 (67,1)
Умеренная (ионизированный кальций сыворотки 1,5–1,79 ммоль/л)	24 (29,3)
Тяжелая (ионизированный кальций сыворотки ≥1,8 ммоль/л)	3 (3,6)

Длительность заболевания на момент включения в исследование составила в среднем 14,2±17,8 мес (медиана — 8,4 мес). У большинства пациентов имелась манифестная форма ПГПТ (72,8%). Умеренная или тяжелая гиперкальциемия выявлена у 33,3% больных. Размеры аденомы околощитовидной железы/желез находились в диапазоне 0,8–6 см. Остеопороз позвоночника/шейки бедра/лучевой кости выявлен у 21 пациента (26,6%), мочекаменная болезнь — у 43 пациентов (54,4%), хроническая болезнь почек (ХБП) ст. 2–4 — у 10 пациентов (12,8%), хроническая сердечная недостаточность — у 18 пациентов (22,2%). Сопутствующая патология имелась у 68,3% пациентов, из их числа у 24 пациентов (42,9%) гастроэнтерологическая патология, у 12 (21,4%) — сердечно-сосудистая патология, у 9 (16,1%) — эндокринная патология (диабет 2 типа, нетоксический зоб), у 5 (9%) — патология почек (пиелонефрит, кисты почек) и другие заболевания. Медиана индекса коморбидности — 1 (диапазон 0–7).

## Изменения качества жизни после хирургического лечения

Медиана длительности наблюдения составила 20 (3–31) мес.

Анализ изменений качества жизни и актуальных симптомов у пациентов в разные сроки после ПТЭ проведен на основании скорректированных средних значений с учетом возможного влияния возраста, формы ПГПТ (манифестная или бессимптомная) и уровня гиперкальциемии (легкая или умеренная/тяжелая), а также исходного качества жизни/выраженности симптомов на динамику показателей.

На рис. 1 представлены скорректированные средние показатели качества жизни по шкалам SF-36 и по суммарному показателю, ФК и ПК опросника PHPQoL у больных ПГПТ до операции, через 3, 12, 24 и более мес после ПТЭ.

**Figure:**
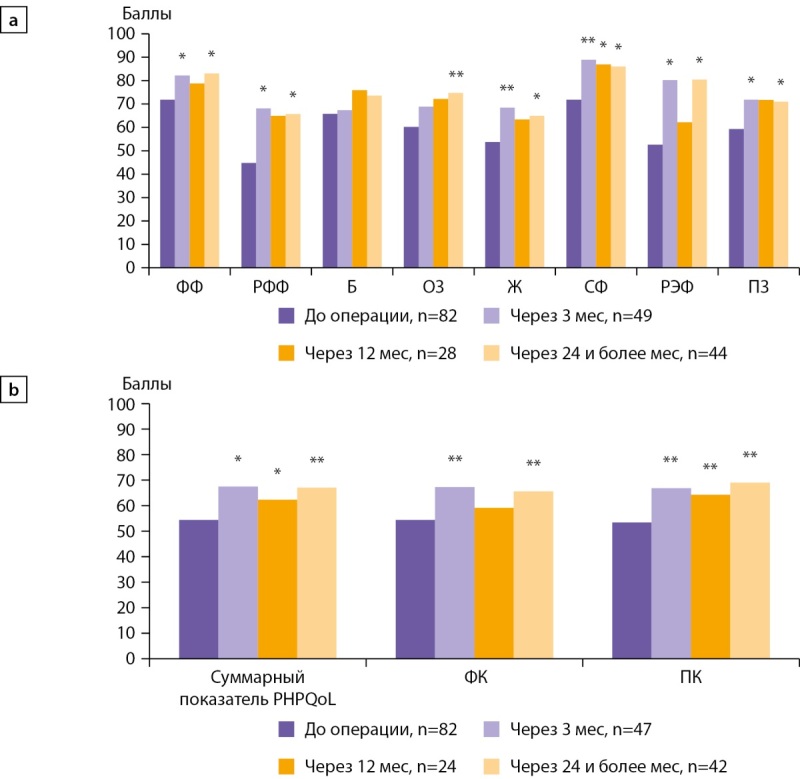
Рисунок 1. Скорректированные по возрасту, форме ПГПТ (манифестная или бессимптомная), уровню гиперкальциемии (легкая или умеренная/тяжелая), а также исходному качеству жизни средние показатели по шкалам опросника SF-36 (а) и по суммарному показателю, ФК и ПК опросника PHPQoL (б) у больных ПГПТ до операции, через 3, 12, 24 и более мес после операции; *p<0,05; **p≤0,001. Шкалы SF-36: ФФ — физическое функционирование, РФФ — ролевое физическое функционирование, Б — боль, ОЗ — общее здоровье, Ж — жизнеспособность, СФ — социальное функционирование, РЭФ — ролевое эмоциональное функционирование, ПЗ — психическое здоровье; ФК —физический компонент качества жизни по PHPQoL, ПК — психический компонент качества жизни по PHPQoL.

До операции наиболее выраженные нарушения качества жизни согласно SF-36 были по шкалам РФФ, РЭФ и Ж (средние значения ≤50 баллов из 100). После ПТЭ имело место статистически значимое увеличение показателей по всем шкалам SF-36, кроме шкалы Б, по сравнению с их предоперационными значениями (GEE, p<0,001). Наиболее выраженное улучшение после операции выявлено по шкалам РФФ и РЭФ (∆=21 и 28 баллов, соответственно). Значимые положительные изменения наблюдали уже через 3 мес после операции по всем шкалам, кроме Б и ОЗ. Как видно из полученных данных, улучшение показателей качества жизни, по сравнению с предоперационными, сохранялось в отдаленные сроки после ПТЭ.

В соответствии с данными специального опросника PHPQoL, у 42,7% больных до операции отмечалось низкое качество жизни (26–50 баллов), а у 2,4% больных — очень низкое качество жизни (0–25 баллов). При этом показатель ФК составил 55,0±18,1 балла, ПК — 52,7±16,5 балла. Через 3 мес после ПТЭ установлено существенное увеличение суммарного показателя PHPQoL (GEE, p<0,05), ФК и ПК (GEE, p<0,001), при этом положительные изменения сохранялись через 12 мес и в отдаленные сроки после операции (GEE, p<0,001), (рис. 1б). Полученные результаты указывают на положительные изменения как специфических для ПГПТ, так и общих аспектов качества жизни пациентов после хирургического лечения.

Отметим, что клинически значимое улучшение качества жизни согласно PHPQoL (увеличение на ≥9 и более пунктов по сравнению с исходным) зарегистрировано у половины (61%) прооперированных больных. Доля больных с клинически значимым улучшением качества жизни сопоставима в группах пациентов с манифестным (62,7%) и бессимптомным течением ПГПТ (59,1%).

До операции актуальными симптомами, которые встречались у более половины пациентов и имели выраженную интенсивность (40–100 баллов), являлись усталость (80%), слабость (64,6%), забывчивость (60,9%), боли в суставах (58,0%), и перемены настроения (50,1%). В разные сроки после операции происходило снижение выраженности данных симптомов (рис. 2).

**Figure:**
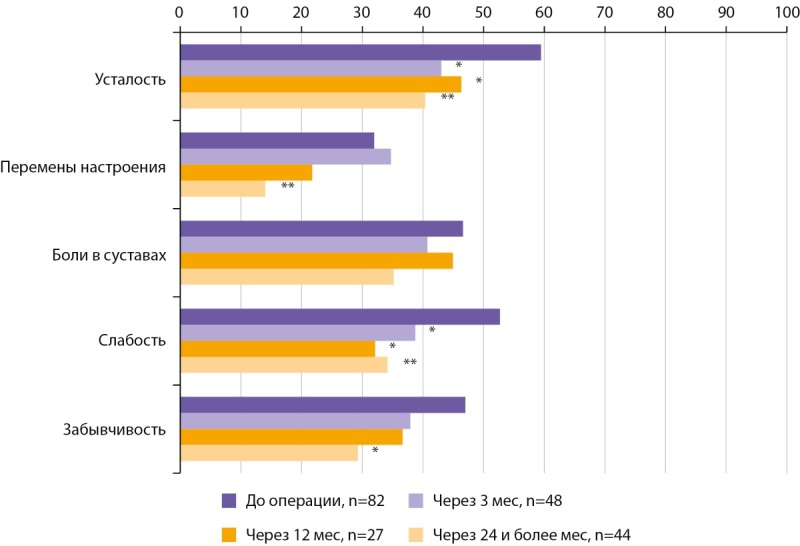
Рисунок 2. Скорректированные по возрасту, форме ПГПТ (манифестная или бессимптомная), уровню гиперкальциемии (легкая или умеренная/тяжелая), а также исходной интенсивности средние показатели выраженности актуальных симптомов у больных ПГПТ до операции, через 3, 12, 24 и более мес после операции; *p<0,05; **p≤0,001.

Как видно из рисунка 2, установлено статистически значимое улучшение (уменьшение выраженности) усталости, слабости, перемен настроения (GEE, p<0,001) и забывчивости (GEE, p=0,005) у пациентов в разные сроки после операции.

Дополнительно проведено сравнение профилей качества жизни у больных ПГПТ по опроснику SF-36 до и в отдаленные сроки после операции (n=57) с профилем качества жизни условно-здоровых респондентов (рис. 3). Средний возраст условно-здоровых респондентов (n=60) 52,5±9,2 года, 95% — женщины. До ПТЭ показатели качества жизни у пациентов были значимо ниже, чем в группе сравнения (p<0,05). В отдаленные сроки после операции показатели по всем шкалам SF-36, кроме РФФ (p=0,011), стали сопоставимы с группой сравнения, что свидетельствует о существенном улучшении и восстановлении общих аспектов качества жизни больных после хирургического лечения.

**Figure:**
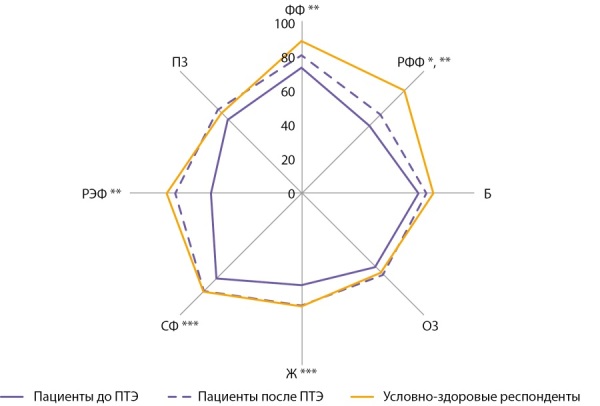
Рисунок 3. Средние показатели по шкалам SF-36 у пациентов до и после ПТЭ в сравнении с условно-здоровыми респондентами; *p=0,011 для сравнения показателей в отдаленные сроки после ПТЭ с показателями условно-здоровых респондентов; ** p<0,001, *** p<0,05 для сравнения показателей до операции с показателями условно-здоровых респондентов.

## Факторы, связанные с улучшением качества жизни больных ПГПТ после хирургического лечения

Перечень анализируемых факторов и результаты однофакторного и многофакторного анализа представлены в таблице 2.

**Table:** Таблица 2. Результаты регрессионного логистического анализа ¹ Зависимая переменная — увеличение суммарного показателя PHPQoL на 9 и более баллов.² Референтная категория.³ Симптом «слабость» не включен в многофакторную модель в виду наличия выраженной его корреляции с симптомом усталость (Spearman r=0,8, p<0,001).

Исходные переменные¹	Однофакторный анализ	Многофакторный анализ
p	ОШ	95% ДИ	p	ОШ	95% ДИ
Возраст, годы	0,075	0,958	0,913–1,004			
Образование Среднее Высшее²	0,477	0,689	0,248–1,92			
Длительность заболевания, нед	0,353	1,004	0,996–1,011			
Исходный Ca²⁺, ммоль/л	0,074	0,044	0,001–1,359			
Исходный ПТГ, пмоль/л	0,310	0,996	0,988–1,004			
Форма ПТГ Бессимптомная Манифестная²	0,766	0,859	0,316–2,336			
Сопутствующая патология Нет Есть²	0,943	1,035	0,398–2,69			
Исходный ФК по PHPQoL	0,023	0,970	0,944–0,996	0,092	1,042	0,993–1,093
Исходный ПК по PHPQoL	0,001	0,936	0,902–0,972	0,004	0,924	0,876–0,976
Исходный уровень усталости	0,002	1,031	1,011–1,052	0,110	1,026	0,994–1,059
Исходный уровень перемен настроения	0,043	1,018	1,001–1,035	0,922	0,999	0,978–1,021
Исходный уровень болей в суставах	0,117	1,010	0,997–1,024			
Исходный уровень слабости³	0,003	1,025	1,009–1,042			
Исходный уровень забывчивости	0,034	1,016	1,001–1,031	0,985	1,000	0,979–1,021

По данным однофакторного анализа, уровень физического и психического компонентов качества жизни по PHPQoL до операции, а также исходный уровень усталости, слабости, изменений настроения и забывчивости являются факторами, связанными со значимым улучшением качества жизни после операции. Принимая во внимание высокие корреляции между исходными уровнями усталости и слабости (r=0,8, p<0,001), в многофакторный анализ включили уровень усталости как более выраженного симптома перед операцией и имеющего более высокое ОШ на этапе однофакторного анализа (1,031 для усталости против 1,025 для слабости).

Из всех включенных в анализ показателей, связанных с пациентом и заболеванием до операции, окончательная многофакторная модель включала только один независимый предиктор значимого улучшения качества жизни после ПТЭ — предоперационный уровень ПК по опроснику PHPQoL (-2 Log=88,5, Nagelkerke R²=0,309; p=0,001). Согласно полученной модели, чем ниже уровень психического компонента качества жизни по PHPQoL до операции, тем больше вероятность значимого улучшения качества жизни по суммарному показателю PHPQoL после операции (ОШ=0,924, 95% ДИ=0,876–0,976, р=0,004).

## ОБСУЖДЕНИЕ

В настоящее время ПТЭ является эффективным методом лечения больных ПГПТ [1, 5, 6]. Для определения эффекта оперативного лечения в условиях пациентоориентированной медицинской помощи важным является учет мнения пациента и оценка изменений качества жизни и спектра симптомов после ПТЭ. Информация о предоперационном уровне качества жизни и траектории его изменения после операции представляет ценность для принятия решений об оптимальной клинической тактике, в том числе, при мягком течении заболевания, когда клинических данных может быть недостаточно в качестве показаний для хирургического лечения.

Ранее нами в рамках промежуточного анализа данных установлены существенные нарушения качества жизни у пациентов с ПГПТ до выполнения ПТЭ и продемонстрированы изменения качества жизни и симптомов после операции [14][15]. Данная работа является продолжением проведенного анализа, выполнена на большем объеме наблюдений и посвящена изучению траектории изменений качества жизни в ранние и отдаленные сроки после ПТЭ и определению степени его восстановления после операции. Также нами изучены факторы, которые связаны со значимым улучшением качества жизни после ПТЭ.

Отметим, что в нашем исследовании, в отличие от многих других работ, была выполнена комплексная оценка качества жизни с помощью сочетания общего опросника SF-36 и специальных опросников — PHPQoL и PAS, что позволило более корректно подойти к оценке влияния болезни и лечения на разные аспекты жизни пациентов. Нами подтверждено, что до операции у больных ПГПТ имелись значительные нарушения качества жизни, более половины пациентов испытывали существенно выраженные усталость, слабость, забывчивость, боли в суставах и перемены настроения. Эти данные соответствует результатам других исследований [8][9][16][17]. При анализе траектории изменений качества жизни после операции нами установлено, что существенное улучшение качества жизни происходило уже через 3 месяца после ПТЭ. Причем положительные изменения сохранялись в течение длительного времени, а именно, при медиане наблюдения после операции 29 мес. Самое выраженное улучшение показателей зарегистрировано по шкалам ролевого физического и ролевого эмоционального функционирования по общему опроснику SF-36. По специальному опроснику PHPQoL установлено значимое улучшение как физического, так и психического компонентов качества жизни после операции. Также показано уменьшение в ранние сроки после ПТЭ интенсивности таких симптомов, как усталость, слабость, изменения настроения и забывчивость с дальнейшим сохранением положительной динамики в отдаленные сроки. Полученные результаты дополняют полученные нами ранее данные мониторинга качества жизни и симптомов после ПТЭ [14][15] и свидетельствуют о стойких длительных положительных изменениях качества жизни больных ПГПТ после операции. Отметим, что выполненный нами анализ изменения качества жизни проведен на основании скорректированных средних с учетом возраста, формы заболевания, уровня гиперкальциемии и исходного качества жизни пациентов.

Известно, что статистически значимые изменения не всегда являются клинически значимыми, т.е. важными для самого пациента. В этой связи нами была определена доля пациентов, у которых наблюдалось клинически значимое улучшение качества жизни. Клинически значимое улучшение качества жизни определяли на основании изменения суммарного балла PHPQoL (≥9 баллов) на любом сроке после операции по сравнению с предоперационным показателем [16]. Примечательно, что более половины пациентов (61%) при длительном наблюдении имели клинически значимое улучшение качества жизни.

Далее нами проведен сравнительный анализ качества жизни больных ПГПТ в отдаленные сроки после операции (более года) и условно-здоровых респондентов, сходных с группой пациентов по полу и возрасту. Оказалось, что по большинству показателей качество жизни больных в отдаленные сроки после ПТЭ было сходным с таковым у условно-здоровых. Данный результат свидетельствует о существенном восстановлении разных аспектов качества жизни больных ПГПТ после операции, их адаптации и возможности вернуться к полноценной жизни.

Наконец, нами изучены предикторы значимого улучшения качества жизни после операции. Информация о факторах, которые связаны с эффектом операции с точки зрения пациента, может способствовать пациент-ориентированному принятию решения о целесообразности хирургического лечения. В настоящее время эти данные мало изучены, что подчеркивает ценность выполненного нами анализа. В перечень факторов, которые были включены нами в регрессионный анализ, вошли возраст, уровень образования, длительность заболевания, уровни Ca²⁺ и ПТГ до операции, форма заболевания, наличие сопутствующей патологии, а также исходный уровень качества жизни и актуальных до операции симптомов. Нами обнаружено, что уровень физического и психологического аспектов качества жизни, а также уровень интенсивности актуальных для ПГПТ симптомов до ПТЭ ассоциированы с улучшением качества жизни в целом после ПТЭ. Все клинические параметры, связанные с заболеванием, и уровень образования не имели прогностического значения для улучшения качества жизни после операции. Единственным независимым фактором, связанным с вероятностью улучшения качества жизни после операции по результатам многофакторного анализа, являлся пониженный показатель психологической составляющей качества жизни до ПТЭ. В целом, полученные данные сходны с результатами недавно опубликованного отечественного исследования [21] и зарубежной работы, выполненной в 2015 году [22]. Так, в работе Ильичевой и соавт. показано, что хирургическое лечение ПГПТ, несмотря на транзиторные осложнения, не мешает улучшению показателей качества жизни при условии достижения ремиссии заболевания, и только персистенция заболевания не позволяет значимо улучшить качество жизни пациентов [21]. В исследовании Ryhänen et al. установлено, что только уровень образования и качество жизни пациента до операции связано со значимым улучшением послеоперационного качества жизни [22]. Согласно нашим данным, чем хуже психологическое состояние пациента до операции, тем более высока вероятность значимого улучшения качества жизни в послеоперационный период. Таким образом, наличие психологических проблем у пациента до операции может рассматриваться в качестве показания для хирургического лечения.

Наше исследование имеет несколько ограничений. Во-первых, включение пациентов происходило только в одном центре. Во-вторых, вследствие дизайна исследования, отсутствовала возможность мониторинга лабораторных показателей после операции. Наконец, имеется некоторое смещение выборки относительно пола (очень мала доля пациентов мужского пола, однако в настоящее время неоспоримо доказано существенное преобладание женщин, больных ПГПТ во всех популяционных исследованиях).

В целом, полученные результаты подтверждают важность учета информации о качестве жизни больных ПГПТ для определения соотношения рисков и пользы при выборе хирургической тактики лечения, а также в процессе наблюдения пациентов после операции для оценки степени их восстановления. Для того, чтобы в полной мере оценить эффективность хирургического лечения на должном уровне, необходимы дальнейшие исследования на большем объеме выборке. Также перспективным является проведение многоцентровых исследований и долговременный анализ качества жизни после ПТЭ совместно с клиническими данными.

## ЗАКЛЮЧЕНИЕ

ПТЭ сопровождается существенным улучшением качества жизни и регрессией симптомов у больных ПГПТ в течение длительного периода после операции. Значимые положительные изменения происходят уже через 3 месяца после ПТЭ и сохраняются при медиане наблюдения 29 мес.

Показатели качества жизни у больных ПГПТ в отдаленные сроки после операции по большинству показателей качества жизни сходны с таковыми у условно-здоровых респондентов.

Независимым предиктором значимого улучшения качества жизни после ПТЭ является предоперационный уровень психологической составляющей качества жизни — чем ниже ее уровень до операции, тем больше вероятность значимого улучшения качества жизни после ПТЭ.

## ДОПОЛНИТЕЛЬНАЯ ИНФОРМАЦИЯ

Источники финансирования. Работа выполнена по инициативе авторов без привлечения финансирования.

Конфликт интересов. Авторы заявляют об отсутствии конфликта интересов.

Участие авторов. Все авторы внесли существенный вклад в проведение исследования и подготовку статьи, прочли и одобрили финальную версию перед публикацией.
